# Bimodal Winter Haul-Out Patterns of Adult Weddell Seals (Leptonychotes weddellii) in the Southern Weddell Sea

**DOI:** 10.1371/journal.pone.0155817

**Published:** 2016-05-19

**Authors:** Lars Boehme, Amy Baker, Mike Fedak, Marius Årthun, Keith Nicholls, Patrick Robinson, Dan Costa, Martin Biuw, Theoni Photopoulou

**Affiliations:** 1 Sea Mammal Research Unit, Scottish Oceans Institute, St Andrews, United Kingdom; 2 British Antarctic Survey, Cambridge, United Kingdom; 3 University of California, Long Marine Laboratory, Santa Cruz, United States of America; Phillip Island Nature Parks, AUSTRALIA

## Abstract

Hauling out is an essential component of pinniped life-history. Haul-out behaviour may be affected by biological (e.g. sex, age and condition) and physical factors (e.g. food availability and environmental conditions), and identifying these factors may help explain the spatio-temporal distribution and habitat use of pinnipeds. The aim of this study is to describe observed winter haul-out patterns of adult Weddell seals in the Weddell Sea and investigate the role of potential predictors to gain insight into the way these animals interact with the physical environment in this region. We examined the haul-out behaviour in relation to available biological (i.e., diving effort, sex) and physical information (i.e., sun angle). Thirty-three satellite telemetry tags were deployed on adult Weddell seals in the southern Weddell Sea during February 2007, 2009 and 2011, following their annual moult recording information on the behavioural mode of the animal: at surface, hauled out or diving. At the end of the austral summer Weddell seals spent, on average, more than 40% of their time hauled out on the ice. Under constant light conditions, it appears that physiological factors drive sex differences in the timing and duration of haul-out behaviour, with females spending on average more time hauled out than males during daylight hours. This time spent hauled-out declined to around 15% in both sexes by the beginning of autumn and remained at this level with a clear nocturnal haul-out pattern during the winter. The time spent diving increased during this period, indicating an increase in foraging effort during the winter months, and led to a common haul-out pattern in both sexes over winter. We found a positive relationship between haul-out duration and the percentage of time spent diving prior to a haul-out in both sexes, with the exception of female daytime haul-outs early in the year.

## Introduction

The Southern Ocean is a dynamic and highly productive marine ecosystem that supports three pinniped species that remain within the pack and fast ice zones year-round (crabeater seal [*Lobodon carcinophagus*], leopard seal [*Hydrurga leptonyx*] and Weddell seal [*Leptonychotes weddellii*]). Weddell seals have been the focus of research for several decades, with studies primarily carried out on fast ice in McMurdo Sound and East Antarctica during austral spring and summer [1–9]. At this time, sea ice extent is decreasing towards its minimum around February and animals are more accessible. However, some studies have focused on their winter ecology [4,10–16]. During the austral winter months seals must gain mass to prepare for the high energetic demands of pupping and breeding from late September [4,17]. Therefore, changes occurring in the environment at this time, e.g., in hydrography, ice cover, productivity, light conditions, and potentially prey abundance and distribution, are likely to be reflected in their behaviour.

While seals spend most of their time in the water, they also haul-out on land or ice to moult, feed their young, rest and avoid aquatic predators, as well as dealing with physiological constraints e.g. thermoregulation [10,13,18,19]. Hauling out is an essential component of their life-history and may be affected by biological (e.g. sex, age and condition) and physical factors (e.g. food availability and environmental conditions) [8,12,19–21]. Most seals that occur at mid-latitudes need to travel between offshore areas where they forage and coastal areas or islands where they can haul-out. As year-round high latitude Antarctic inhabitants, Weddell seals have an almost constant opportunity to haul-out on fast ice, pack ice or sea ice at, or near to areas where they forage.

Identifying the physical and biological factors affecting haul-out behaviour may help explain the spatio-temporal distribution and habitat use of Weddell seals, for which foraging areas and haul-out areas need not be geographically distant. Assuming equal accessibility [22], patterns of habitat use are thought to be largely influenced by prey resources [23] or in other terms, the spatio-temporal distribution outside of the breeding season is thought to be driven by the availability of prey. For example, dive duration and bottom times for elephant seals increased when foraging in areas of low prey quality [24] and ringed seals showed longer dives and shorter surface intervals when increasing their foraging effort [25]. Harbour seals have been found to change their activity patterns including haul-out behaviour in relation to the quality, availability and spatial distribution of their prey, when foraging occurs close to their haul-out sites [26]. These correlations may even be more pronounced in Weddell seals compared to other species, as they can often haul-out where they successfully forage. Haul-out and diving behaviour should therefore be dominated by Weddell seals’ physiological constraints and prey availability and we would expect to see increased foraging and diving and less time spent hauled out when prey is scarce or of lesser quality. Under this assumption, investigating these two behaviours ought to provide information regarding the prey resources animals are exploiting. Although detailed dive data were not available in this study, we used the timing and duration of haul-outs, together with summary dive data, to make inferences about the haul-out behaviour of Weddell seals over winter and investigate seasonal changes therein. Time spent engaged in one activity must be traded off against another, so by investigating temporal trends in haul-out behaviour we can gain insights into animals’ seasonal activity budgets.

As upper trophic level predators, seals are often associated with areas of enhanced productivity [4,14,27,28] and in some cases, these areas have been found to be essential to reproductive success and population growth [13,21,29,30]. However, the logistical challenges of measuring productivity and, the generally low chlorophyll values in the Southern Ocean, make detecting such signals difficult. Instead, as explained above, variation in the Weddell seals’ behaviour may reflect changes in prey resources and might be used to make inferences about patch quality, availability and spatial distribution of the prey [20,25,26,31].

In recent decades, technological advancements in animal-borne instruments have made it possible to collect and transmit data from remote locations [13,14,32–34], enabling the collection of data throughout the austral winter in the Southern Ocean. Such instruments are able to record information on the behaviour of the animal and sometimes also the *in situ* physical environment and transmit stored records to the ARGOS satellite system [33,35,36]. These integrated datasets can be used to examine the ecological responses of animals to changes in the marine environment [3,27,36,37].

**Fig 1:**
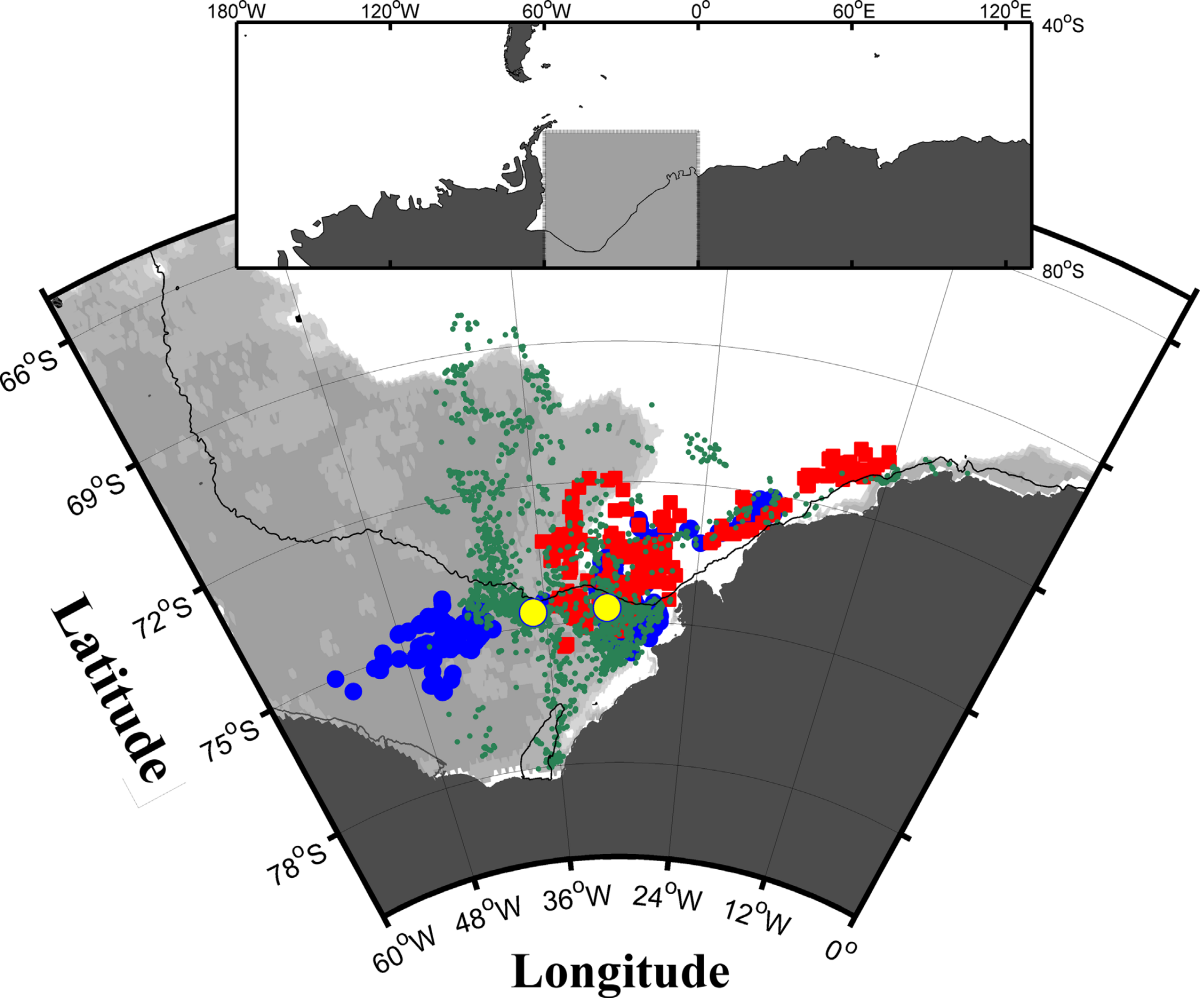
Map of the Weddell Sea showing the haul-out locations. Haul-out locations of 31 Weddell seals in the southern Weddell Sea, Antarctica during the austral winter of 2007 (n = 4, blue circles), 2009 (n = 8, red squares) and 2011 (n = 19, dark green dots). For the purposes of this study the black contour line depicts the continental shelf break at 1000 m depths and the dark grey patch is the Antarctic continent including ice shelves. The yellow circles highlight the two tagging sites used in all years. The inset figure at the top shows the study area (lighter grey) in the wider context. Bathymetry data are from the ETOPO1 model [39]. Light grey shaded areas correspond to an observed sea ice concentration of more than 20% on 1^st^ March 2011; based on daily AMSR-E/Aqua sea ice concentration data on a 12.5 km grid [40].

Previous studies have described a strong diurnal haul-out pattern for Weddell seals during the austral spring and summer, with seals foraging at night in response to the diel vertical migration of prey, and hauling out mostly during the day [1,10]. Haul-out events were then found to shift from a diurnal cycle to a nocturnal cycle during austral autumn and winter when seals primarily hauled out at night [9,10,19,20]. The mechanisms driving this shift are currently unknown, but changes in pinniped haul-out patterns appear to be influenced by the seasonal variation in physical environmental variables (e.g., air temperature, solar radiation, wind speed, sea-ice cover, tide and time of day) [4,5,7,10,14,38] and prey resources [9,19]. In this study, we investigate biological and physical factors, which might drive the winter haul-out behaviour of Weddell seals across the continental shelf break in the southern Weddell Sea (Fig 1), including the continental shelf, continental slope and offshore waters (68 to78°S, 8 to 50°W).

The Weddell Sea is a deep embayment formed along the coast of the Antarctic continent, topographically bordered to the west by the Antarctica Peninsula. Heavy sea ice conditions occur year round, with floating ice from the Filchner-Ronne Ice Shelf extending over the continental shelf [41]. The cyclonic gyre in the Weddell Sea causes upwelling of nutrient rich waters at the continental shelf break [41], increasing productivity in this area. Although the distribution and movement of Weddell seals are expected to be governed by the availability of breathing holes, cracks and leads, the timing and duration of haul-out events may provide some insight into the complex interactions of seals with the marine environment [12,13,19,42].

The aim of this study is to describe observed winter haul-out patterns of Weddell seals in the Weddell Sea and investigate the role of potential predictors to gain insight into the way animals exploit and interact with the physical environment in this region. This is addressed by examining the haul-out behaviour in relation to biological (diving effort prior to a haul-out, sex, individual variability) and physical factors (sun angle as a function of season and light availability).

## Materials and Methods

### Ethics statement

The capture and tagging protocols have been reviewed and approved by the University Teaching and Research Ethics Committee (UTREC) and the Animal Welfare and Ethics Committee (AWEC) as part of our ethical review process and was scrutinized under the UK Animal (Scientific Procedures) Act 1986. Capture and deployment of satellite transmitters was carried out by experienced personnel with UK Animal (Scientific Procedures) Act 1986 Personal Licenses.

### Data collection and processing

Thirty-three satellite telemetry tags were deployed on adult (lengths between 210 and 250 cm) Weddell seals in the southern Weddell Sea on both sides of the Filchner Depression during February 2007, 2009 and 2011, following their annual moult (Fig 1). Seals were sedated with an intramuscular injection of Zoletil (0.2–0.4 mg kg^-1^) based on visual assessment of the approximate weight of the seal [42,43], and morphometric measurements and biological samples were collected [43]. Each seal was equipped with a Conductivity-Temperature-Depth Satellite Relay Data Logger (CTD-SRDL), attached to the head or upper neck of the animal using a two-part, rapid-setting epoxy resin [44,45]. SRDLs were designed and manufactured at the Sea Mammal Research Unit Instrumentation Group, St Andrews, UK and CTD sensors constructed at Valeport Ltd, Devon, UK [33]. Once deployed, the tags were expected to fall off no later than during the following year’s moult.

The CTD-SRDLs were equipped with wet-dry and pressure sensors to monitor, process and record information on the behavioural mode of the animal: at surface, hauled out or diving [36]. A dive was recorded when the wet-dry sensor was wet and below 6 meters for more than 8 seconds. A haul-out began when the wet-dry sensor had been dry for 10 minutes and the event ended when the sensor was wet for 40 seconds or when the pressure sensor recorded the seal was deeper than 6 meters for 8 seconds [36]. All time not hauled-out and not diving was considered to be at the surface. For each 4-hour period the percentage spent in these three modes was recorded including the average dive depth and time. CTD-SRDL design and programming are described in more detail by Boehme et al. [33] and data collection, compression and transmission techniques discussed by Fedak et al. [36].

The start and end times (in UTC) of each haul-out were also collected and processed. All data were stored for transmission via the ARGOS satellite system. When a transmission was received by the ARGOS system, the geographic locations of stored records were estimated using the Doppler effect [35]. Location estimations were generally good as most transmission were received by the ARGOS system when seals were hauled-out on the ice. More than 50% of locations were estimated from 4 or more transmissions and were assigned an ARGOS location class of 0 or better. We therefore assume that most location estimates have a standard deviation of less than ±5 km [46,47]. After a seal had been hauled out for 16 hours the tag automatically ended the event and began another. Such breaks in haul-out intervals were removed and the haul-outs joined when the end time of one haul-out equalled the start time of the subsequent haul-out. Each haul-out’s time stamp was corrected by its associated longitude to obtain local time. The local start and end times for each haul-out were then used to calculate the time of the centre of the haul-out (Tm) and the haul-out duration (Td). For each haul-out, the mean of the percentage time spent diving within the two 4-hour summary periods incorporating the haul-out start time and the preceding 4-hour period were used to obtain a measure of ‘dive effort’ (De).

Of the 33 tagged seals, data from 2 seals were excluded from the data analysis. These two tags did not provide accurate time stamps for haul-out events and therefore haul-out duration could not be calculated. Tags fail for a number of reasons including seal death, instrument malfunction, battery exhaustion and tag loss [10]. CTD-SRDL tags provided data for an average of 163 days in 2007, 76 days in 2009 and 187 days in 2011. Details of tag deployment are summarized in Table 1. This resulted in a dataset of 4,856 haul-out events from 31 seals.

**Table 1:** Summary of CTD-SRDL deployments on Weddell seals in the Weddell Sea during the austral winters of 2007, 2009 and 2011. Data from the two highlighted deployments were excluded from the dataset.

Seal ID	Nr	Sex	PTT	First haul-out	Last haul-out	Deployment length in days	
ct27-W1-07	1	F	22484	12-Feb-07	09-Oct-07	239	
ct27-W2-07	2	M	28487	12-Feb-07	23-Jul-07	161	
ct27-W3-07	3	M	43856	13-Feb-07	02-Oct-07	231	
ct27-W5-07	4	F	43863	12-Feb-07	04-Mar-07	20	
ct-43-059-09	5	F	92137	14-Feb-09	26-Feb-09	12	
ct-43-574-09	6	F	92144	07-Feb-09	27-Mar-09	48	
ct-43-582-09	7	F	92138	08-Feb-09	24-Mar-09	45	
ct-43-613-09	8	F	92136	07-Feb-09	14-Jun-09	128	
**ct-43-858-09**	**9**	**x**	**48929**	**05-Feb-09**	**14-Feb-09**	**9**	
ct-43-860-09	10	F	48928	05-Feb-09	09-Apr-09	63	
ct-43-862-09	11	M	43844	05-Feb-09	17-Sep-09	224	
**ct-43-864-09**	**12**	**x**	**43841**	**06-Feb-09**	**15-Feb-09**	**10**	
ct-43-865-09	13	M	43840	05-Feb-09	10-Mar-09	33	
ct-43-866-09	14	F	43839	05-Feb-09	30-Mar-09	54	
ct70-356-11	15	M	43861	08-Feb-11	23-Feb-11	15	
ct70-486-11	16	M	43875	09-Feb-11	02-Aug-11	174	
ct70-488-11	17	F	43876	10-Feb-11	08-Oct-11	240	
ct70-490-11	18	M	43871	09-Feb-11	28-Sep-11	231	
ct70-491-11	19	M	43859	15-Feb-11	16-Oct-11	244	
ct70-499-11	20	M	43880	11-Feb-11	29-Sep-11	230	
ct70-500-11	21	M	48928	11-Feb-11	29-Sep-11	230	
ct70-501-11	22	F	48921	08-Feb-11	05-Oct-11	240	
ct70-502-11	23	F	43881	10-Feb-11	27-Mar-11	46	
ct70-503-11	24	F	48922	08-Feb-11	16-Sep-11	220	
ct70-526-11	25	M	43841	11-Feb-11	31-May-11	110	
ct70-633-11	26	M	43839	14-Feb-11	19-Sep-11	217	
ct70-634-11	27	M	43844	11-Feb-11	13-Jul-11	153	
ct70-637-11	28	F	22490	14-Feb-11	04-Oct-11	232	
ct70-638-11	29	F	43846	09-Feb-11	23-Aug-11	195	
ct70-640-11	30	F	43840	12-Feb-11	29-Oct-11	259	
ct70-642-11	31	F	1545	11-Feb-11	03-May-11	81	
ct70-643-11	32	F	43850	12-Feb-11	29-Oct-11	260	
ct70-650-11	33	F	22483	10-Feb-11	27-Jul-11	167	
Average deployment duration							146

### Environmental data

Although air temperature and wind speed have been shown to influence the haul-out behaviour of seals [10,48], accurate data were not available within the study area. The nearest weather station, Halley Research Station (British Antarctic Survey, UK), was more than 250 km from the majority of haul-out events. The pack ice the seals inhabit is also marked by pressure ridges. Therefore, air temperatures and wind speeds interpolated from this station to haul-out locations or large scale atmospheric analyses (e.g. NCEP) are unlikely to provide an accurate representation of atmospheric conditions at the seal’s location on the ice and were not used in the data analysis. While tides may play a role for the haul-out behaviour for some pinnipeds, tidal currents in the study region are generally low off the shelf with typical current speeds of less than 5 cm/s. On the shelf speeds are higher, but still less than 30 cm/s. The effect on the water surface elevation is also generally less than 50 cm [49]. We therefore assumed that tides will not affect the haul-out events and diving behaviour of the Weddell seals in this region.

Light conditions were *a priori* considered to be the most influential to haul-out events and foraging patterns. We therefore calculated the angle of the sun above the horizon based on the location and time in UTC using formulas developed to time astronomic observations [50]. We also created a binary variable that bisected light conditions into effectively light (civil twilight from dawn to dusk) and effectively dark (nautical twilight from dusk to dawn). Information on cloud cover was not used in this study, as it was not available on this small scale.

### Statistical analysis

As part of exploratory data analysis we plotted a histogram of the raw data for haul-out duration, which revealed a clear bimodal pattern. We used the k-means function in R [51] to identify the mean values of the two clusters observed in the response variable, haul-out duration. One cluster had a mean duration of 1.06 hrs (Standard Error (SE) of 0.0003, n = 3587) and the other 10.82 hrs (SE 0.0027, n = 1269). We therefore classified the haul-outs in this study into two haul-out types: short duration haul-outs, and long duration haul-outs. Haul-out events that were less than three hours in length we classified as short haul-outs (n = 3338), and those that were longer than six hours were classified as long haul-outs (n = 1266). Haul-outs between three and six hours in length were excluded from the analysis (n = 252). The estimated groupings were checked for consistency against empirical estimates identified visually during exploratory data analysis.

Pairwise plots revealed that relationships between the response and explanatory variables were non-linear. In addition, multiple haul-out events were recorded for each individual seal, so we modelled haul-out duration as a binary variable (long (0) vs short (1)) using a Generalized Additive Mixed Model (GAMM), with the “mgcv” package in R [52,53]. We fitted the models using the “gam” function together with the “re” smooth basis function to fit random effects. In addition, the relationships between the response and explanatory variables were found to be different for each of the sexes, so a separate model was fitted to clustered response data from males and females.

The candidate explanatory variables used were timing of the haul-out (hour of the day at the centre of the haul-out period), the percentage of time spent diving in the two preceding 4-hour summary periods, year, a variable for darkness or daylight and individual seal ID. Timing, time spent diving and light conditions were fitted as fixed effects and individual ID as a random effect. Model selection was carried out manually using the AIC and the proportion of deviance explained.

We were interested in finding out if the timing of the haul-out could be used to explain its duration, however, the timing of haul-out events changed over time, during the course of the deployments. To accommodate this time-dependence, we fitted day of the year as a two-dimensional smooth with timing of the haul-out on a 24hr scale (0–23). We did not find a significant difference between deployment years, which could be the result of the dataset dominated by one year (2011). We therefor did not include deployment year in the model. Both binary and five-point ordinal darkness scales were explored as explanatory variables but neither was important for either males or females when the two-dimensional smooth was included. This was assessed based on the change in model deviance and the flatness of the estimated smooth relationships. The two-dimensional smooth was fitted using the “te” basis function, the percentage of time spent diving was fitted using a “cs” shrinkage basis function [54], and the random effect for individual was fitted using the “re” smooth for simple random effects [53,55]. The number of knots for each smooth was selected according to methods suggested in the “mgcv” package manual and [52]. The model’s roughness penalty (the “gamma” parameter in the gam function) was set to 1.4 to reduce the chance of over-fitting and reduced maximum likelihood (REML) was chosen as the smoothing parameter estimation method [53]. The model form for both sexes was as follows:

$${\mathrm{haul out duration}}_{\mathrm{ij}}\;=\;f_{1}(\mathrm{day of the year},\;\mathrm{time of day})\;*\;{\mathrm{day of the year}}_{i}\;*\;{\mathrm{time of day}}_{i}+f_{2}(\mathrm{time spent diving})\;*\;{\mathrm{time spent diving}}_{i}+f_{3}(\mathrm{individual})\;*\;{\mathrm{individual}}_{j}+{\mathrm{epsilon}}_{\mathrm{ij}}$$

where f_1_ is a tensor product of day of the year and time of day, f_2_ is a smooth function with shrinkage for percentage of time spent diving prior to a haul-out, and f_3_ is a function for implementing a simple random effect for individual seal. Epsilon is the error term which has a binomial distribution.

## Results

### Observational results

**Fig 2:**
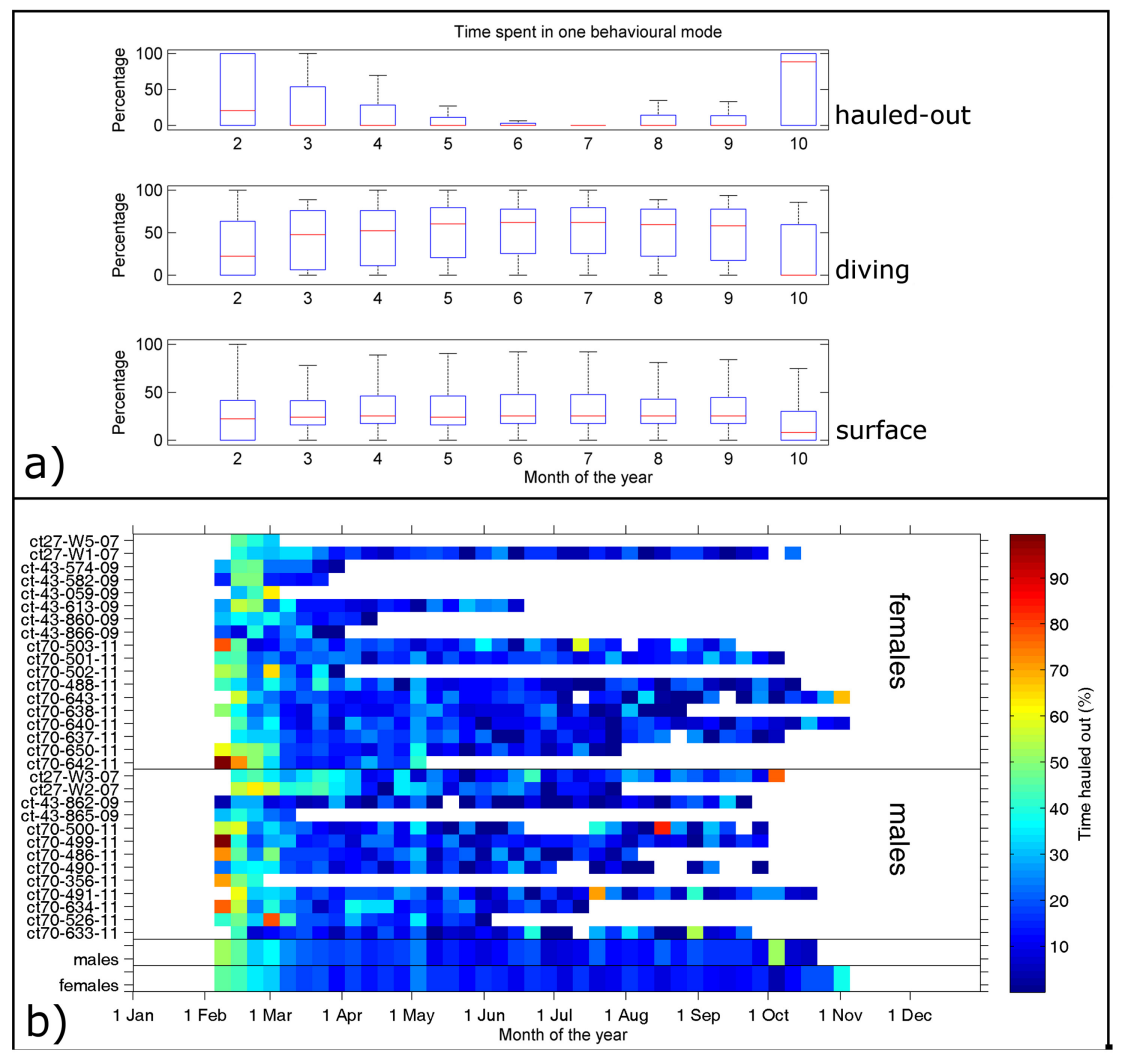
Time series of time spent in different behavioural modes. (A) Time series of monthly percentage time spent in different behavioural modes for all seals. (B) Weekly average of percentage time spent hauled out for each seal used in this study. The two bottom lines show the weekly mean values for males and females respectively.

Overall 74% of all haul-out events identified by the SRDLs were received and archived (S1 File). However, there was a strong temporal effect, with more than 90% of all haul-out information received in February to April and less than 50% from July onwards (S1 Fig). The data later in the deployment were not presumed to be representative of the haul-out behaviour and we therefore focus on the timeframe from February to mid-winter in June. Most haul-out events occurred close to the shelf break on both sides of the Filchner Trough, where most seals were tagged (Fig 1). The overall percentage of time hauled out per day was 18% (4.32 hrs) with standard deviation of 9% (2.16 hrs), but this varied seasonally. Seals spent, on average, more than 40% (9.60 hrs) of their time hauled out on the ice after their annual moult (Fig 2A), but this percentage quickly declined to around 15% (3.60 hrs) by the beginning of March and remained below this level for the rest of the deployment, with no significant interannual variation (Fig 2B). The time spent diving rose from a median of about 22% in February to about 47.5% in March. It then increased slightly to about 60% around May and stayed relatively constant, while time spent at the surface was constantly between 22 and 26% (Fig 2A). While there is individual variation, all animals showed the same overall pattern (Fig 2B). The two average time series of weekly percentage of time spent hauled out for males and females (Fig 2B) were highly correlated even when reducing the degrees of freedom (R^2^ = 0.76, p<10^−10^) indicating that there are no significant differences in the temporal change in the amount of time spent diving, at the surface and hauled-out between males and females.

**Fig 3:**
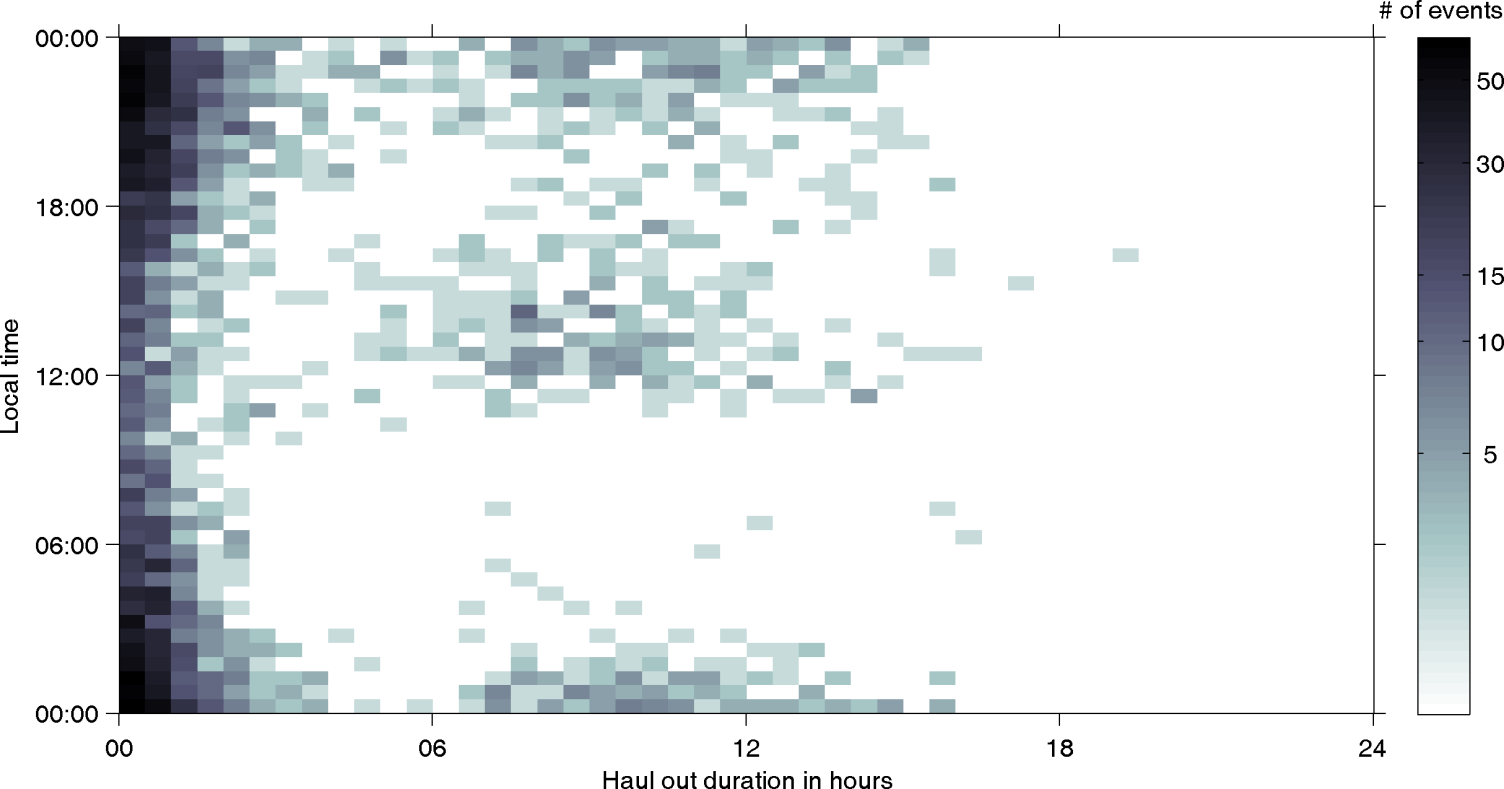
Logarithmic density plot of haul-out timing over duration. Number of haul-out events over the centre time of haul-outs (Tm) and duration of haul-outs (Td) for the complete dataset. The darker the grid cell the more events occurred (see colour bar).

**Fig 4:**
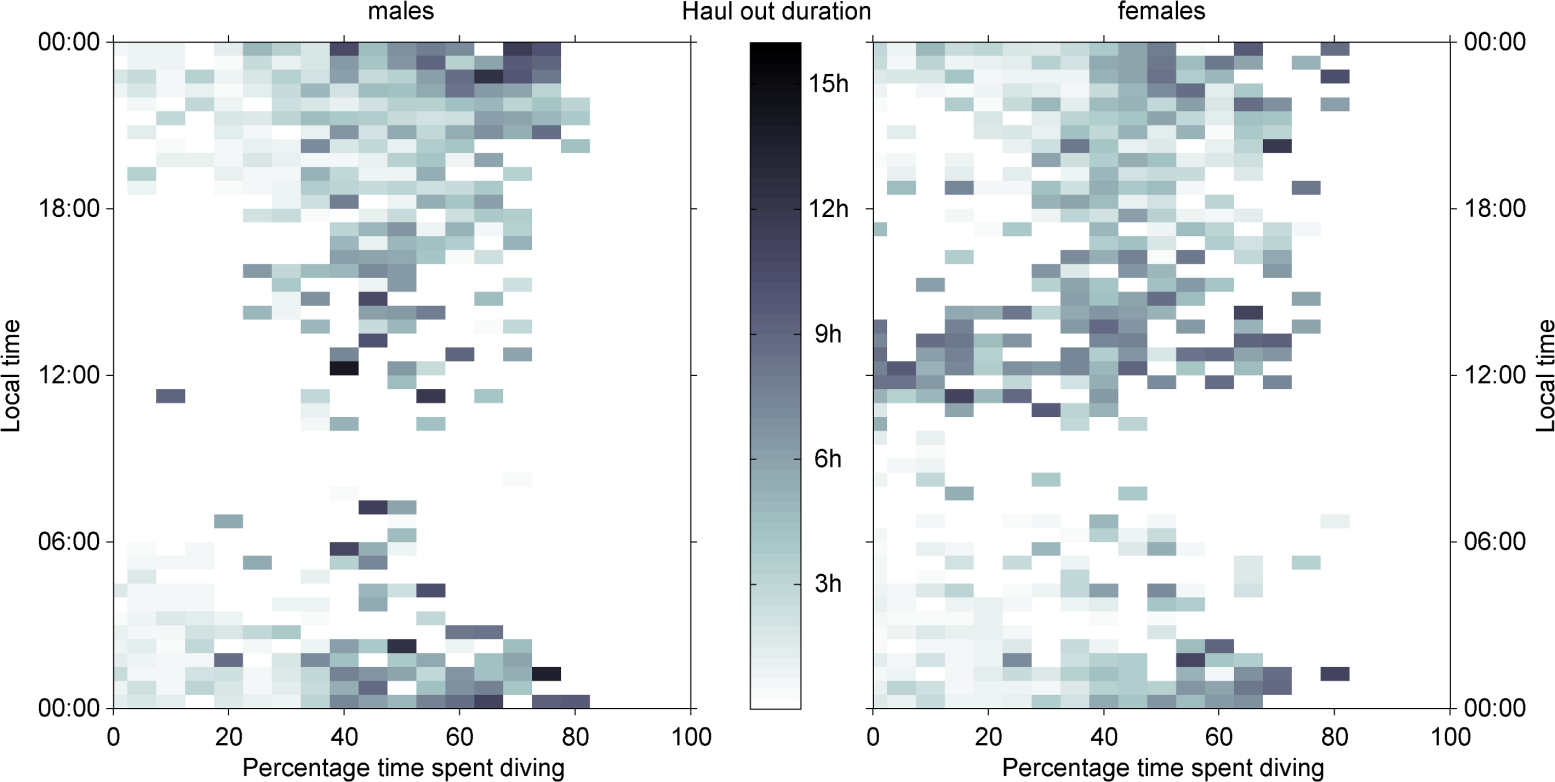
Timing of haul-out event vs. diving effort. Local time of the centre of haul-out events in relation to the diving effort preceding that event, as defined in the text (Tm vs Td) for males (left) and females (right). Only boxes with at least 2 haul-outs are shown. The darker the colour of the grid cell the longer the average haul-out duration corresponding to that percentage of diving.

Haul-out behaviour was found to have a 2-dimensional bimodal pattern according to duration and the time of day at the centre of the haul-out. About 69% of all haul-outs were short (<3 hours), while about 25% of haul-outs were longer than 6 hours (Fig 3) confirming the exploratory statistical data analysis. The overall ratio of short to long haul-outs was 2.75 to 1. This was consistent for females (2.71 to 1) and males (2.79 to 1). Short duration events occurred throughout the entire day, and the majority were nocturnal (Fig 3). About 60% of all long duration events occurred between 18:00 and 06:00 local time (night) while 33% of long duration haul-outs occurred between 11:00 and 17:00 local time (Fig 3). The latter, daytime long haul-outs, were mainly carried out by females (Fig 4 and S2 Fig). We found that the duration of daytime haul-outs cannot be explained by diving effort in the preceding 4 hours, while haul-outs during the night show a strong correlation with the percentage time spent diving previous to the haul-out (Fig 4 and S3 Fig).

**Fig 5:**
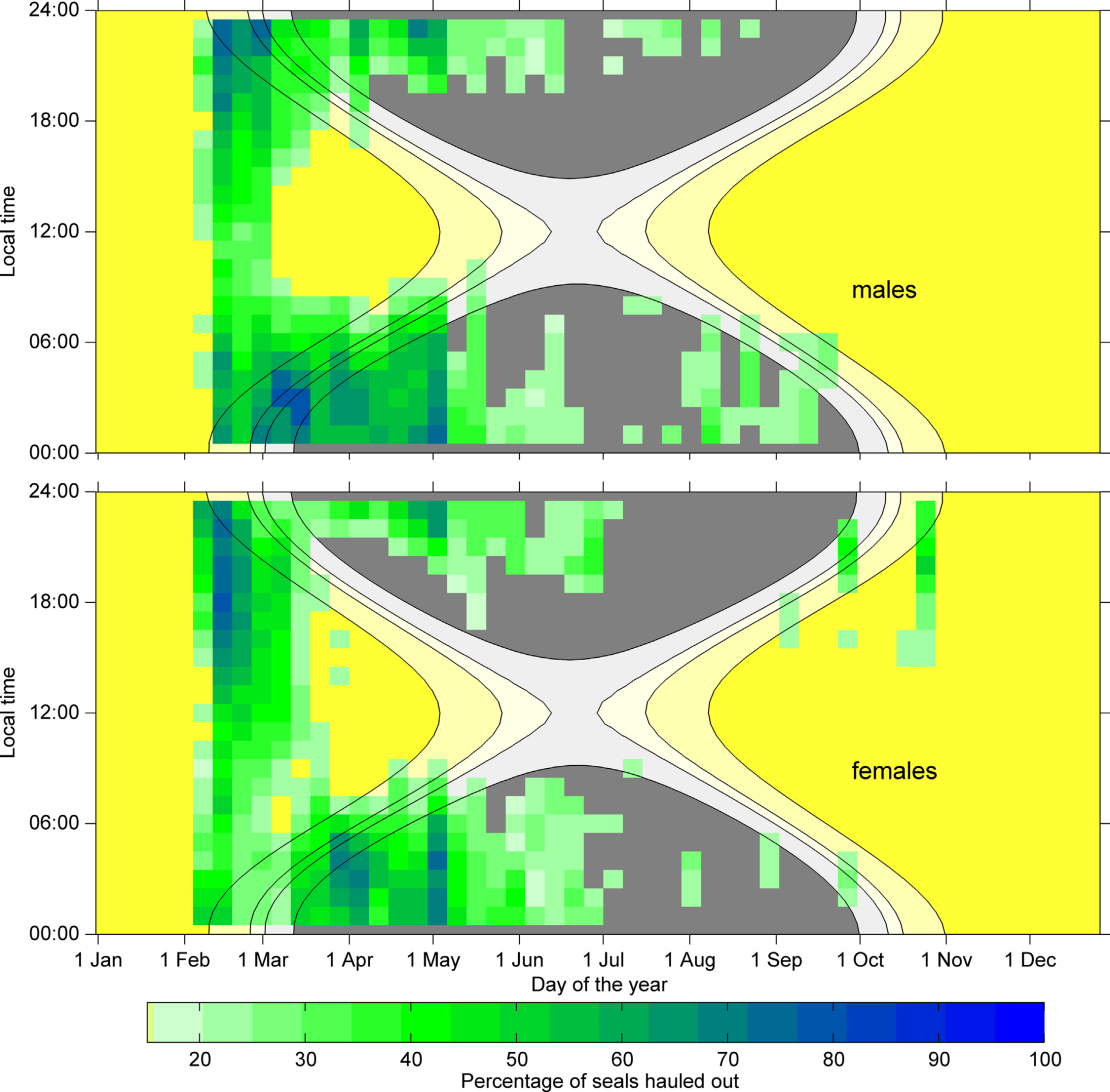
Density plot of percentage of seals hauled out within the specific hour. Densities for males (top) and females (bottom) of less than 20% are not shown. Background colours show the sun angle for an observer at latitude of 74.5°S with contour lines for astronomical twilight (-12°), nautical twilight (-8°), civil twilight (-6°) and sun rise/set (-0.8°). Night time (sun angle below -8°) is grey, while day time (sun angle above -8°) is yellow.

To investigate the change in haul-out behaviour over time we looked at the timing of haul-out events (Tm) in relation to the time of day and day of year, which together act as a proxy for light availability. The timing of haul-outs was found to be different for males and females in the early phases of the deployment with no differences between years (Fig 5).

At the beginning of the deployments (Feb-Mar) the light conditions are such that there is constant daylight. At this time of year, male Weddell seals spent more than 50% of their time hauled out between 13:00 and 06:00 local time, while spending more time (>50%) in the water during the morning and noon time (Fig 5). As soon as the seals were able to ‘choose’ between times with daylight or darkness (early March, ca. day 59), male seals shift to mainly hauling out during the hours of darkness and spent more than 80% of their time in the water during the daylight hours.

Female Weddell seals showed a slightly different pattern early in the deployments. Under constant light conditions they spent more than 50% of their time hauled out between 13:00 and 01:00 local time, while spending more time (>50%) in the water during the early morning between 01:00 and 10:00. They also spent more than 40% hauled-out between 10:00 and 13:00 (Fig 5), while males were mostly diving. These haul-outs mainly consist of long haul-outs that are not driven by diving effort (Fig 4). Females also increased their time spent diving around midday much later in the season than males, in late March (ca. day 79), to more nocturnal haul-outs. From late March to the end of the deployment period, females and males showed similar nocturnal haul-out behaviour (Fig 5).

When the daylight starts to diminish even around local noon (early June, ca. day 152) less than 20% of male and female seals were hauled out at any given time (Fig 5). We did not find a drift in the timing of haul-out events on a day to day basis supporting our assumption that tides do not play an important role.

### Modelling results

**Fig 6:**
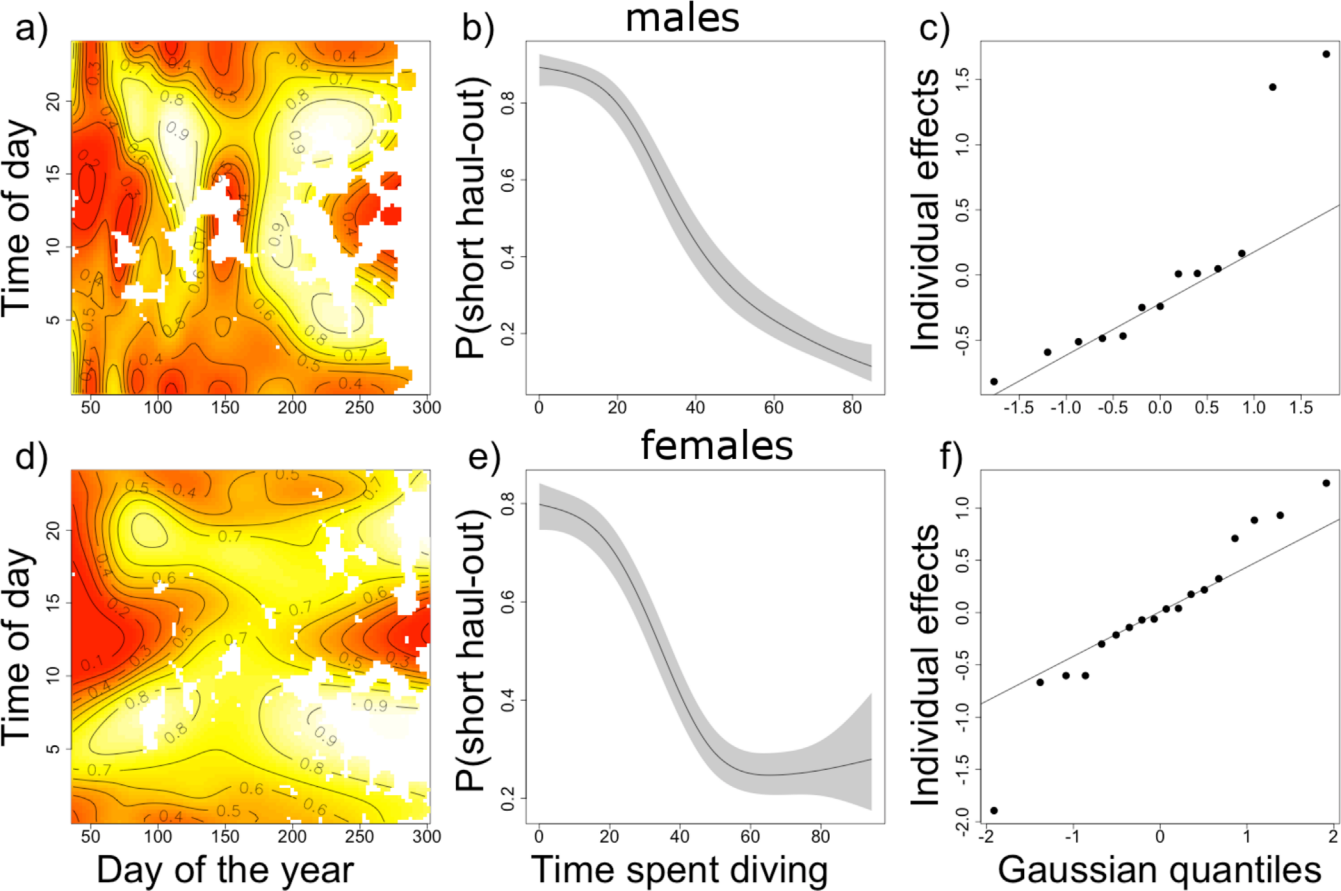
Marginal relationship between model covariates and the probability of a short haul-out. The first column shows the estimated relationship for the tensor product between time of day and day of the year. Areas of low probability of short haul-outs appear red and areas of high probability of short haul-outs appear yellow. The black contour lines give the estimated probability of short haul-out events across the two-dimension space shown. The second column shows the smooth relationship between time spent diving prior to a haul-out event and the probability of a short-duration haul-out. The third column shows the random effect for individual. The relationships are shown for males (top row, figures a to c) and females (bottom row, figures d to f).

The fitted relationship between the response and each of the explanatory variables is shown in Fig 6 for males (a-c) and for females (d-f). There was a clear diurnal pattern in haul-out duration for both sexes, and this pattern varied seasonally. Early in the year seals hauled out for longer in the middle of the day, between the hours of 10:00 and 15:00 local time, and around midnight. This pattern was especially clear in females and more diffuse in males. The midday peak in haul-out duration disappeared around day 100 and reappeared between day 200 and 250. There was a positive, monotonic relationship between haul-out duration and the percentage of time spent diving prior to hauling out in male Weddell seals (Fig 6). In females, the relationship was positive until 60% time spent diving in the previous 4 hours and then levelled out between 60% and 90%, with increased uncertainty (Fig 6). There was greater individual variability in the female responses than in the males (Fig 6C and 6F). Lastly, the models explained 30% and 31% of the variability in the data for females and males, respectively (deviance explained).

## Discussion

This is the first time haul-out behaviour of Weddell seals has been investigated in the Weddell Sea. We found a previously unobserved 2-dimensional bimodal pattern in haul-out duration with respect to time of day at the centre of the haul-out. Furthermore, this pattern differed seasonally between males and females. Our results suggest that there is a sexual segregation in the timing and duration of haul-out events during austral summer and autumn, but not in the weekly percentage of time spent hauled out. The daily distribution of haul-outs was found to be different for males and females during the autumn, but the sexual segregation in haul-out behaviour started to break down as early as the beginning of March. The disappearance of this diurnal pattern coincides with a change in seasonal light availability. We suggest that this difference in allocation of time spent hauled out by males and females represents different haul-out strategies. These might be driven by diving behaviour, sex-specific energetic or life-history requirements such as the early establishment of underwater territories in males, energetics relating to implantation in females, or increased energy conservation in females in the period following the moult. If so, then we would expect these differences to be more pronounced during times when males and females have different life-history objectives, e g. during the pupping and breeding time.

In this study, the daytime haul-outs, occurring early in deployment, showed no correlation with the diving effort in the preceding 4 hours, especially for female Weddell seals. Instead, these diurnal haul-outs may be driven by sex-specific and life history requirements while foraging effort is reserved for the times of day when prey is more abundant and easier to capture. Weddell seals moult at the end of summer (December through to March) and the increased haul-out duration could be related to minimising the energetic cost of growing new hair and taking advantage of the increased radiation from the sun and higher air temperatures in the time after noon to maintain the best skin temperature for the moult [56,57]. The increased ratio of females that display these long haul-outs suggests that female Weddell seals in the study region moult later than the male population and potentially later than some other populations along the Antarctic coast line [9,57]. Female Weddell seals especially at the southern end of their distribution often do not gain enough stores over the winter time to support lactation in early spring explaining the need for a mixed income-capital breeding strategy [57,58]. The increased time at the surface during the day could therefore be a strategy to trade-off environmental help with thermoregulation to finish the annual moult with starting to improve body-composition by feeding.

Previous studies have described a strong diurnal haul-out pattern during the austral spring and summer. Weddell seals were found to haul-out at night and forage during the day in response to the diel vertical migration of prey [1,10,13]. We have not found such a clear pattern early in the deployment, which we attribute to the availability of light throughout the day. However, we observed a developing diurnal pattern early in the deployment, at the end of the summer, with most time hauled out during the darker hours of the day and increased time in the water in the mornings (Fig 5). We have shown a clear relationship between the diving efforts preceding the haul-outs around local midnight (Fig 4) confirming the findings of previous studies assuming that increased diving effort is related to foraging. This diurnal pattern became stronger during the winter, when the available light was reduced or disappeared completely, depending on latitude. The diurnal pattern found in the present study early in the year was analogous to that of Weddell seals in eastern Antarctica [10], crabeater seals in the Antarctic Peninsula [12,13] and harbour seals on Svalbard [48], which experience a similar seasonal light regime, although the seasonal variation differed. Hamilton et al. [48] showed a daily rhythm in haul-out behaviour for harbour seals on Svalbard when light and darkness were both available, as well as under constant light conditions, but not during the polar night when there was no light available. The main difference between their results and the results of this study is that Weddell seals also displayed a diurnal pattern under full dark conditions. Nevertheless, harbour seals on Svalbard experience ‘complete’ darkness, while most Weddell seals in this study still experience some brightness even at mid-winter. This might prevent the collapse of diel vertical migration in prey in the Weddell Sea, which might then be reflected in seal foraging behaviour.

During austral winter, sea ice extends over much of the Weddell Sea, covering 99% of the study area (Fig 1). This provides constant availability of suitable haul-out habitat. Resting on a solid substrate appears to be essential to many species, where long, multi-day foraging trips begin and end on land [42,59], while others, such as elephant seals, spend months at sea without hauling out, instead carrying out drift dives for resting [60,61]. Russell et al. [62] show that grey and harbour seals around the UK split the overall time spent resting between resting on land and resting at sea. In this study, the time spent hauled out decreases in winter. This observation is consistent with previous observations [10] and was also shown for other seal species experiencing reduced light availability [13,48]. The total weekly haul-out duration began to decrease early on in the deployment period, from more than 40% to less than 15% by end-March, and stayed low until the end of the winter (Fig 2). One explanation for this decrease would be that this is a thermoregulation strategy. At high latitudes air temperature can be tens of degrees Celsius colder than sea temperature, and lengthy haul-outs may lead to thermal stress. Given the results of this study, the findings of Hamilton et al. [48] and the fact that resting at sea, on the surface or at depth, is common in other species, it seems possible that seals living at high latitudes switch to spending more time resting under or in water when air temperatures drop below some threshold and light availability decreases. Our data seem to support this argument, as we see a decrease in time spent hauled-out with the advance of winter (Fig 2A). However, the seals do not exchange the reduced haul-out time with time spent at the surface but instead spend more time diving. The move from hauling-out on the ice to spending more time in the water can therefore not only be a thermoregulation strategy, but might also mean the need for increased foraging. Such a change in the diving behaviour could be the result of prey resources getting smaller with the advance of the winter [16]. Female Weddell seals in particular are thought to be not able to find enough food during the winter to regain mass and body condition after breeding and moulting as well as supporting gestation [57]. Our findings suggest that Weddell seals allocate a large amount of time during the winter to diving, i.e. foraging.

A strong correlation was found between night time haul-outs and the percentage of time spent diving in the 4 hours prior to the haul-out. Weddell seals are opportunistic, visual predators that use the under-ice surface to backlight prey when foraging [63], therefore shifting dive effort to the time of day when light is available, would be beneficial in detecting and capturing prey. Seals may also be using the available daylight to perform exploratory dives, looking for alternative breathing holes, cracks, or leads, or to locate areas associated with new prey patches. Weddell seals consume a number of benthic and pelagic prey species including fish, cephalopods, and invertebrates [63,64]. In the Weddell Sea, *Pleuragramma antarcticum* (Antarctic silverfish) is the most abundant species and believed to be the main prey of Weddell seals [1]. *P*. *antarcticum*, a pelagic schooling fish, aggregates in the water column based on life cycle stage and time of day, vertically migrating in relation to light intensity [1,16] where it may be foraging on zooplankton [10]. Changes in prey behaviour, influenced by bottom-up trophic dynamics, would be reflected in predator foraging strategies and in foraging effort. Weddell seals need to alter their foraging behaviour to maximize foraging success and focus their effort at times when prey is more abundant or easier to capture. Therefore, the shift we observed in the timing of haul-out events, from throughout the day to a clear diurnal pattern, and the strong correlation between percentage of time spent diving prior to the night time haul-outs and the reduction of time spent hauled-out in the winter may be best explained as a response to seasonal variation in prey resources (e.g., prey availability and distribution). A recent study has found a reduction in *P*. *antarcticum* abundance related to ocean warming along the Western Antarctic Peninsula [65]. *P*. *antarcticum*’s early life history is tightly linked to sea ice [66] [67] and changes in the sea ice concentration and extend may have profound impacts on the availability of this main prey and therefore on the Weddell seals. However, further analysis and the inclusion of individual diving data are needed to examine how seals use their underwater environment in this region.

The biggest hindrance in studying the Weddell seal population in the pack ice of the southern Weddell Sea is the remoteness of the region and the inability to retrieve animal-borne instruments that can collect large amounts of data. More sophisticated instruments are needed that have the ability to analyse the data internally before transmitting data using telemetry. This would enable us to go from inferring e.g. foraging from dive data or changes in the haul-out behaviour to recorded feeding events [68]. Nevertheless, we were able to show that the shift in haul-out behaviour between austral summer and austral winter is evidence that seals are responding to the seasonal variation in their environment [10,12,13,69]. Because foraging success is critical to their fitness and reproductive performance, prey availability and distribution are expected to be driving factors in the timing and duration of haul-outs. Therefore, changes in the hydrographic features that alter prey behaviour, distribution or availability, will influence the foraging strategies and diving behaviour of the seals and thus the haul-out patterns [10,69]. In addition to that, we posit that these effects manifest differently in males and females under constant light conditions, when intrinsic factors may be dominating, but that the harsh thermal conditions override these differing intrinsic drivers during the winter months at high latitudes, reflected in the common haul-out patterns observed over winter.

## Supporting Information

S1 Fig Histogram showing the percentage of haul-out events received with regards to all haul-out events recorded by the tag per month. (TIF)

S2 Fig Histogram of timing of long haul-out events (6h < Td <16h) for male and female Weddell seals. (TIF)

S3 Fig Relationship between haul-out duration and percentage time spent diving preceding the haul-out for males (left) and females (right) separated into haul-outs at night (top) and day (bottom). (TIF)

S1 File Haul-out dataset collected by Weddell seals as used in this study. (CSV)
