# Supplementary figures and images for: Bimodal Winter Haul-Out Patterns of Adult Weddell Seals (Leptonychotes weddellii) in the Southern Weddell Sea
Source: PLoS One. 2016 May 19;11(5):e0155817. doi: 10.1371/journal.pone.0155817 (PMC4873014; doi:10.1371/journal.pone.0155817)

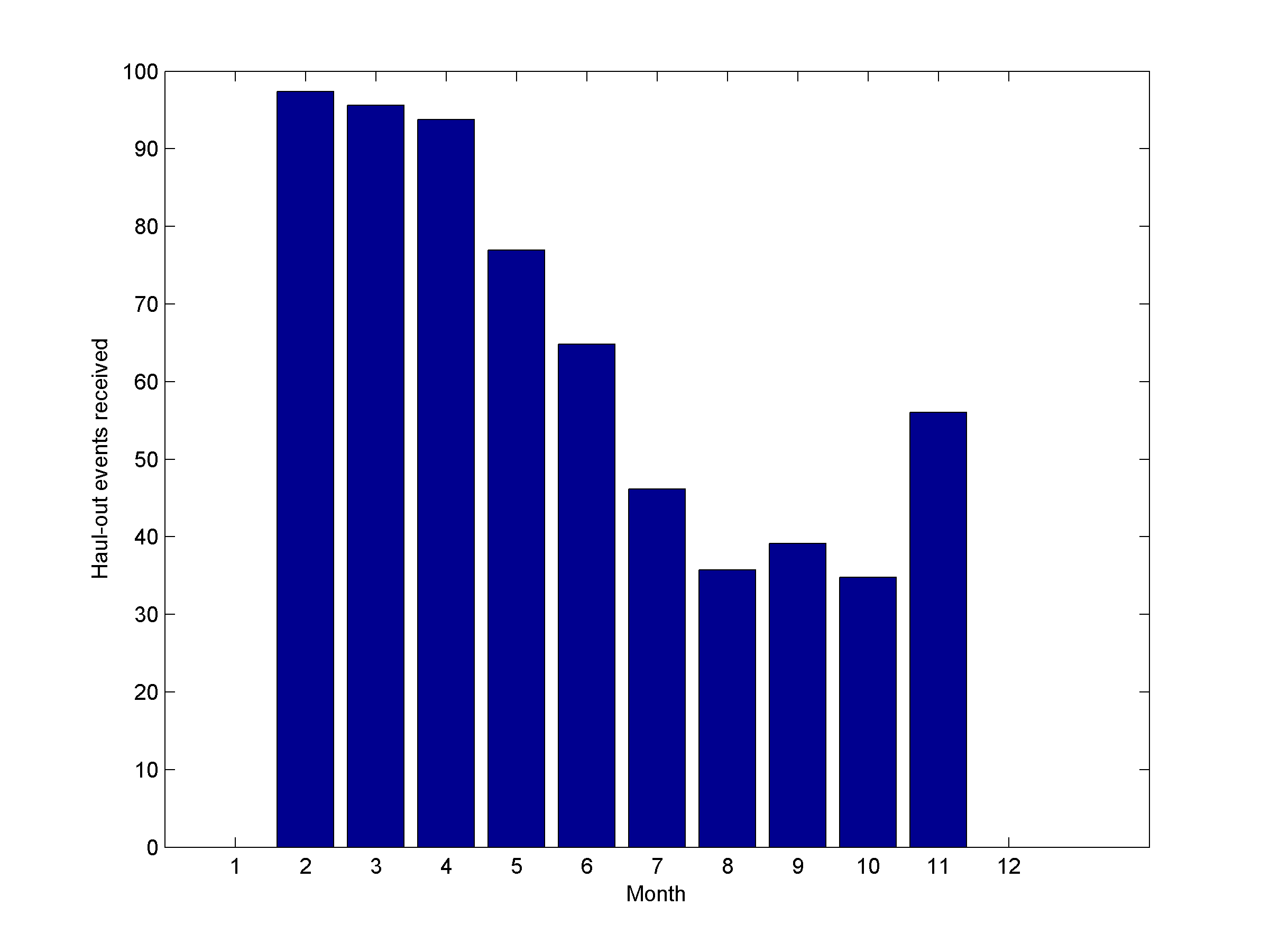

Supplement: S1 Fig — (TIF) [file pone.0155817.s001.tif]

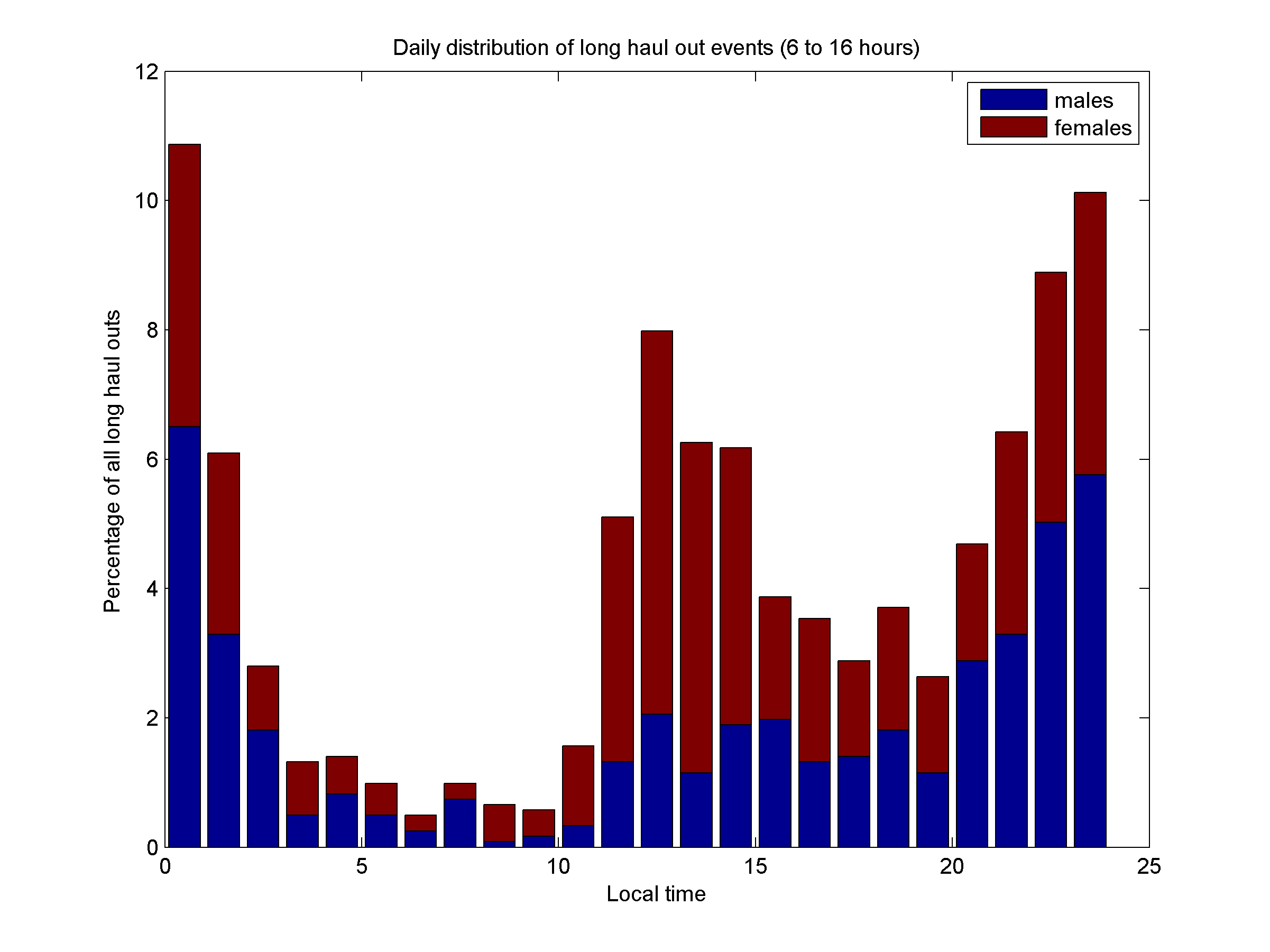

Supplement: S2 Fig — (TIF) [file pone.0155817.s002.tif]

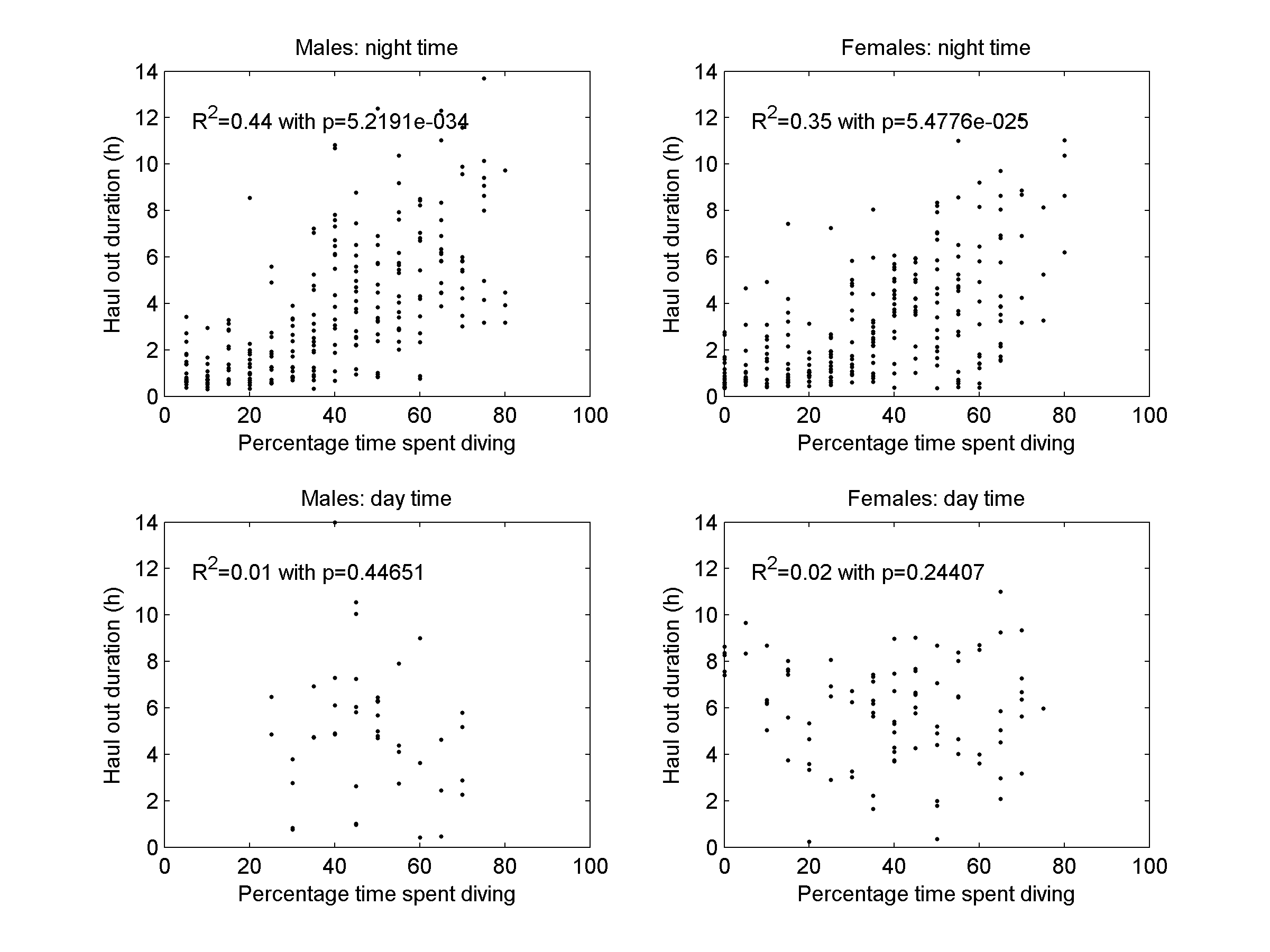

Supplement: S3 Fig — (TIF) [file pone.0155817.s003.tif]
